# Comparison of translation loads for standard and alternative genetic codes

**DOI:** 10.1186/1471-2148-10-178

**Published:** 2010-06-14

**Authors:** Stefanie Gabriele Sammet, Ugo Bastolla, Markus Porto

**Affiliations:** 1 Institut für Festkörperphysik, Technische Universität Darmstadt, Hochschulstr. 8, 64289 Darmstadt, Germany; 2 Centro de Biología Molecular 'Severo Ochoa', (CSIC-UAM), Cantoblanco, 28049 Madrid, Spain; 3 Karlstr. 23, 64283 Darmstadt, Germany; 4 Institut für Theoretische Physik, Universität zu Köln, Zülpicher Str. 77, 50937 Köln, Germany

## Abstract

**Background:**

The (almost) universality of the genetic code is one of the most intriguing properties of cellular life. Nevertheless, several variants of the standard genetic code have been observed, which differ in one or several of 64 codon assignments and occur mainly in mitochondrial genomes and in nuclear genomes of some bacterial and eukaryotic parasites. These variants are usually considered to be the result of non-adaptive evolution. It has been shown that the standard genetic code is preferential to randomly assembled codes for its ability to reduce the effects of errors in protein translation.

**Results:**

Using a genotype-to-phenotype mapping based on a quantitative model of protein folding, we compare the standard genetic code to seven of its naturally occurring variants with respect to the fitness loss associated to mistranslation and mutation. These fitness losses are computed through computer simulations of protein evolution with mutations that are either neutral or lethal, and different mutation biases, which influence the balance between unfolding and misfolding stability. We show that the alternative codes may produce significantly different mutation and translation loads, particularly for genomes evolving with a rather large mutation bias. Most of the alternative genetic codes are found to be disadvantageous to the standard code, in agreement with the view that the change of genetic code is a mutationally driven event. Nevertheless, one of the studied alternative genetic codes is predicted to be preferable to the standard code for a broad range of mutation biases.

**Conclusions:**

Our results show that, with one exception, the standard genetic code is generally better able to reduce the translation load than the naturally occurring variants studied here. Besides this exception, some of the other alternative genetic codes are predicted to be better adapted for extreme mutation biases. Hence, the fixation of alternative genetic codes might be a neutral or nearly-neutral event in the majority of the cases, but adaptation cannot be excluded for some of the studied cases.

## Background

The origin and universality of the genetic code is one of the biggest enigmas in biology [1]. Soon after the genetic code of *Escherichia coli *was deciphered [2], it was realized that this specific code out of more than 10^84 ^possible codes is shared by all studied life forms (albeit sometimes with minor modifications). The question of how this specific code appeared and which physical or chemical constraints and evolutionary forces have shaped its highly non-random codon assignment is subject of an intense debate. In particular, the feature that codons differing by a single nucleotide usually code for either the same or a chemically very similar amino acid and the associated block structure of the assignments is thought to be a necessary condition for the robustness of the genetic code both against mutations as well as against errors in translation [3-13]. This robustness reduces fitness losses due to mutation and mistranslation, which is believed to be a major force in coding sequence evolution [14]. There are three basic theories of the genetic code's nature, origin, and evolution. Whereas the stereochemical theory first proposed by Gamow [15] asserts that the codon assignment was originated by the physicochemical affinity between the amino acid and the codon or anticodon, the adaptive theory posits that the genetic code was shaped under selection for robustness, either against mutations [16,17] or against translation errors [18,19], or against both [18,20,21]; finally, the coevolution theory postulates that the structure of the genetic code reflects the pathways of amino acid biosynthesis [22].

In this work, we address an issue that has received somewhat less attention in this broader context, namely the existing variants of the standard genetic code. These variants are used for instance in the mitochondria of many species, and they consist in the modification of one or several codons of the standard genetic code. Two main mechanisms have been proposed to explain how they may have evolved despite the large fitness cost that is expected to be associated with the modification of a codon [9,23,24]: through an ambiguous intermediate state and through the reassignment of a temporarily unused codon. These mechanisms are not mutually exclusive. The first one assumes that a codon is temporarily recognized both by the current as well as by a mutated tRNA, so that it can result in two different amino acids. Such ambiguity might be preferential in some circumstances and remain present for some time. A codon reassignment occurs if the mutated tRNA finally takes over. The second scenario takes place if one codon disappears from a given genome. This is particularly likely in small genomes with large guanine/cytosine (GC) or adenine/thymine (AT) content, as it is the case for many mitochondrial genomes and nuclear genomes of parasitic microbes. In this case the translation system may change without any cost, and the codon may be 'conquered' by another amino acid. Both scenarios consider that alternative genetic codes are the result of non-adaptive or neutral evolution, even though 'genomic streamlining' (i.e., selective pressure to minimize the genome by eliminating a tRNA) has been proposed as a possible advantage of code changes [25,26]. However, it has not been addressed whether these variants differ from the standard genetic code as far as mutation and translation loads are concerned.

Here, we use computer simulations of neutral protein evolution constrained to maintain the folding and misfolding stability of the native state in order to study the differences between the standard genetic code and seven naturally occurring variants concerning their effects on protein stability. These effects are predicted using a simplified model of protein folding [27], the same that we consistently use in the evolutionary simulations [28-32]. Despite its simplicity, this model is able to predict with similar accuracy as other more complicated models the effects of mutations on folding stability. Due to our simplifying assumption to consider a neutral model, the different genetic codes hardly have any influence on the average unfolding and misfolding stabilities. However, alternative codes yield significantly different mutation and translation loads, in particular for genomes evolving under strong AT or GC mutation bias.

## Results and Discussion

In this work, we study how the genetic code influences the fitness consequences of errors (loads) during mutation and translation. This influence may arise because of two mechanisms: (1) Directly, through the change in the rate of occurrence of different amino acid misincorporations in the translated protein; (2) Indirectly, through the evolutionary influence that the genetic code may have on protein stability. We simulated our previously proposed model of protein evolution in order to study this indirect influence as well.

## Model

Our model of protein evolution has been presented in previous works [28-32], and it is similar to models used by others [33-40]. It has been successfully used to explain non-Poissonian rates in neutral evolution [28] and the observed site-specific amino acid distributions [31,32], to name two examples. It considers a genetically homogeneous population, i.e. the product of the population size *N *and the mutation rate *μ *is assumed to be small. The assumption of a small mutation rate *μ *is justified when considering an individual protein, but not an entire genome. If we considered a whole evolving genome instead of a single protein, the approximation of very small mutation rate would not be justified, since genomic mutation rates are in a range of 0.003 to 0.004 mutations per genome per generation for DNA-based microbes, including viruses, bacteria, and eukaryotes [41]. In this context, a new interesting effect has to be considered, namely the hitch-hiking effect, which consists in the fixation of mildly disfavorable alleles driven by a positively selected allele present in the same chromosome. However, considering the hitch-hiking effect would make the study much more complicated, and we leave it as a subsequent step. In our model, the fitness of an individual carrying a particular gene depends only on the folding properties of the translated protein, which are estimated through a simple protein folding model. A characteristic of our model that distinguishes it from similar ones is that we consider two types of stability, with respect to misfolding and with respect to unfolding. They are calculated by estimating the normalized energy gap α(**A**) and the folding free energy *F*(**A**), respectively. Misfolding stability is measured through *α *and unfolding stability is measured through *-F*, which are computed for each protein sequence **A **encountered in the simulated evolution. The protein structure is assumed to have been already optimized by natural selection and is kept fixed throughout evolution, as represented by the experimental structure found in the Protein Data Bank (PDB) (see Methods). If the folding stability is too small, the protein will not be stable in its native state; if the misfolding stability is too small, misfolded structures can trap the folding process, and they can expose hydrophobic patches and promote aggregation. In the spirit of Kimura's neutral theory of molecular evolution [42,43], we assume that mutations are either neutral or strongly deleterious. More specifically, all proteins having both unfolding and misfolding stabilities above previously fixed thresholds are regarded as viable and they are assigned the same fitness ℱ = 1 (in arbitrary units) and all proteins for which at least one of the stabilities is below threshold are regarded as unviable and they are assigned fitness ℱ = 0. Mutations to stop codons are considered lethal and receive a fitness ℱ = 0. The two neutral thresholds *α*_thr _and *F*_thr _are chosen proportional to the values of *α*_nat _and *F*_nat _of the respective protein in the PDB, multiplied with coefficients slightly smaller than one so that the native protein is above threshold. We present results with both coefficients equal to 0.98, but our results are robust to changing this prefactor in a reasonable range. Note that fitness functions depending continuously on stabilities can be considered, but the resulting non-neutral evolutionary dynamics is significantly more complex due to population size effects [44]. Neutrality of the fitness landscape is assumed here for the sake of simplicity, since otherwise the model would depend on at least two additional parameters, i.e. the smoothness of the fitness landscape and the effective size of the population, making it very difficult to reach clear conclusions about the effect of the genetic code and the mutation bias.

**Figure 1:**
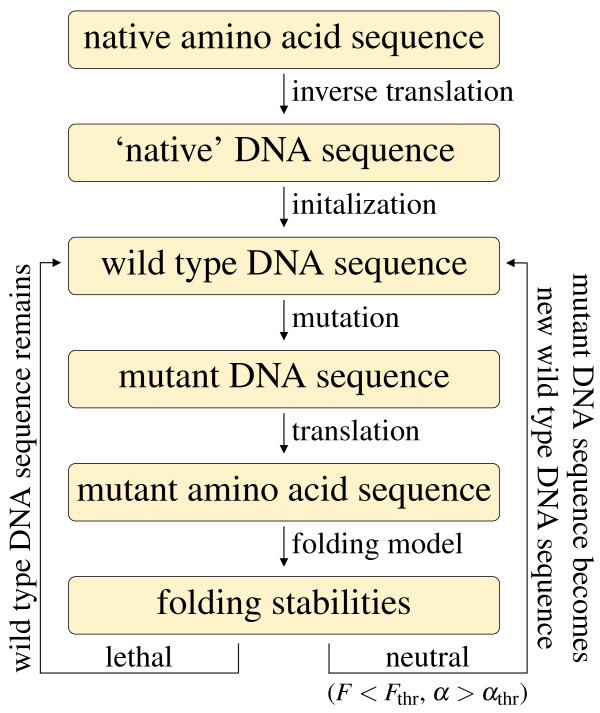
**Model**. Sketch of the model: We start each simulation with the native amino acid sequence as obtained from the Protein Databank (PDB) of the chosen structure, from which we construct a corresponding 'native' DNA sequence by randomly choosing codons using the genetic code under consideration with weights determined by the given AT content (inverse translation). This 'native' DNA sequence is hence as close as possible to the equilibrium with the chosen AT content and becomes the first wild type DNA sequence. Then, at every step, the current wild type DNA sequence is mutated to generate a mutated DNA sequence, which is translated to a mutated amino acid sequence using the genetic code under considerations. The resulting mutated amino acid sequence is evaluated using the folding model with respect to its folding stabilities, based on which the mutated amino acid sequence is either considered as neutral (both unfolding and misfolding stability are above threshold) and the mutated DNA sequence becomes the new wild type DNA sequence, or as lethal (one or both stabilities are below threshold) and the mutated DNA sequence is discarded (the wild type DNA sequence remains as is).

**Figure 2:**
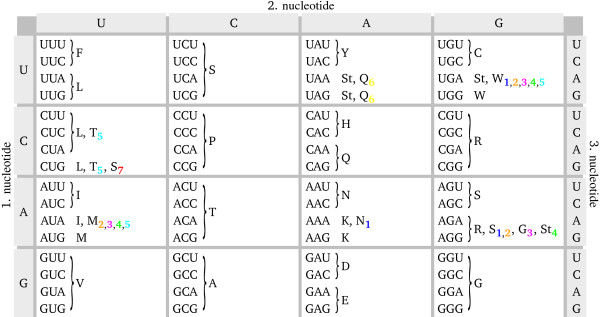
**Standard genetic code and naturally occurring variants**. The standard genetic code and the naturally occurring variants studied in this work, written using the RNA alphabet and standard abbreviations for the amino acids ('St' indicates stop codon). Concerning the alternative genetic codes, we follow the naming scheme of the NCBI database (http://www.ncbi.nlm.nih.gov/Taxonomy/Utils/wprintgc.cgi, including the transl_table numbering). We give in a following only a very brief description of a given alternative genetic code's systematic range, a more detailed description with references can be found on the NCBI's web page. The standard genetic code is shown in black (3 stop codons), whereas the seven naturally occurring variants studied are shown using the following color scheme: (1, blue): 'The Echinoderm and Flatworm Mitochondrial Code' (NCBI transl_table=9), mitochondrial code of Asterozoa, Echinozoa, and Rhabditophora (4 differences, 2 stop codons) [48]; (2, orange): 'The Invertebrate Mitochondrial Code' (NCBI transl_table=5), mitochondrial code of Nematoda, Mollusca, Crustacea, and Insecta (4 differences, 2 stop codons) [48]; (3, magenta): 'The Ascidian Mitochondrial Code' (NCBI transl_table=13), mitochondrial code of Urochordata (4 differences, 2 stop codons) [48]; (4, green): 'The Vertebrate Mitochondrial Code' (NCBI transl_table=2), mitochondrial code of Vertebrata (4 differences, 4 stop codons) [49]; (5, cyan): 'The Yeast Mitochondrial Code' (NCBI transl_table=3), mitochondrial code of *Saccharomyces cerevisiae, Candida glabrata, Hansenula saturnus, and Kluyveromyces thermotolerans *(6 differences, 2 stop codons) [50]; (6, yellow): 'The Ciliate, Dasycladacean and Hexamita Nuclear Code' (NCBI transl_table=6), nuclear code of Ciliata, Dasycladaceae, Diplomonadida (2 differences, 1 stop codon) [51]; (7, red): 'The Alternative Yeast Nuclear Code' (NCBI transl_table=12), nuclear code of *Candida albicans *(1 difference, 3 stop codons) [52]. The color scheme is as in Figs. 3 to 6.

Another important ingredient of our model is the mutation model at the DNA level. We parameterize the mutation model with a single parameter, the AT bias, which represents the equilibrium content of adenine and thymine after a very long evolution under mutation alone (the complementary variable GC bias, expressing the equilibrium content of guanine and cytosine under mutation alone, is sometimes alternatively used). The mutation bias strongly affects the substitution process (i.e., the accepted mutations), biasing the amino acid composition of the protein. Interestingly, the mutation bias also influences the folding properties of the evolving proteins [32,45]. In fact, AT rich codons code for amino acids which are more hydrophobic and the resulting proteins tend to be more stable against unfolding (more negative folding free energy *F*) but less stable against misfolding (since the set of all potential misfolded protein structures increases their stability faster than the native structure, resulting in a smaller normalized energy gap *α*), whereas the contrary holds in case of GC bias. This bias at the mutation level produces a bias at the substitution level, both for neutral fitness landscapes [32] and for smooth fitness landscapes [44], such that proteins evolving under higher AT bias will be comparatively more stable against unfolding but less stable against misfolding. Finally, in order to fully specify the mutation model, we have to fix the transition-to-transversion ratio *k*. Since transitions (such as C to T) tend to conserve the physiochemical properties of the coded amino acid more than transversions (such as C to A or to G), a high transition-to-transversion ratio *k *usually reduces the mutation load. We used two values of k, *k *= 2, which is suitable for most nuclear sequences [46] and a kind of standard value in molecular evolution simulations, and *k *= 20, a maximal value that has been observed in some mitochondrial genomes [47]. To study the influence of the mutation process, we simulate the evolution of DNA sequences under nine different mutation biases with both transition-to-transversion ratios *k *= 2 and *k *= 20, Our model of protein evolution cannot be treated analytically, so that we have to study it using numerical simulations (see Fig. 1). Point mutations change the DNA sequence (see Methods) and they are accepted if the resulting amino acid sequence, translated from the DNA sequence using the genetic code under consideration, is viable, i.e. both stabilities are above threshold. We simulated the evolution of three different proteins of similar lengths and different secondary structure compositions, (i) the epsilon subunit of F1F0-ATP synthase (PDB id. 1aqt, chain A, 135 amino acids), (ii) the acyl carrier protein (PDB id. 1hy8, chain A, 76 amino acids), and (iii) the cold-shock protein (PDB id. 1c9o, chain A, 66 amino acids), see Methods for details. We start each simulation with the native amino acid sequence as obtained from the PDB of the chosen structure, from which we construct a corresponding 'native' DNA sequence by randomly choosing codons using the genetic code under consideration with weights determined by the given AT content (inverse translation). The influence of this starting sequence is lost after a relatively short evolutionary trajectory needed for equilibration, after which a stationary situation is reached, in which both stabilities fluctuate around constant values. Statistics is taken only in this stationary state. We compare the standard genetic code with seven naturally occurring variants, see Fig. 2. Out of these seven variants, five are used in mitochondria of many species, and two variants are used by certain species in their nuclear protein production. These variants differ in between one to six codon assignments, and display between one to four stop codons instead of the three stop codons of the standard code, cf. Fig. 2. We follow the naming scheme of the NCBI database http://www.ncbi.nlm.nih.gov/Taxonomy/Utils/wprintgc.cgi, including the transl_table numbering). To ease the assessment of our results, we use a consistent color scheme in this work, in which the standard genetic code is shown in black, whereas the naturally occurring variants are color coded according to the following scheme:

1.Blue: 'The Echinoderm and Flatworm Mitochondrial Code' (NCBI transl_table = 9) [48]; Taxonomic range: Asterozoa (starfishes), Echinozoa (sea urchins), Rhabditophora among the Platyhelminthes.

2 Orange: 'The Invertebrate Mitochondrial Code' (NCBI  transl_table = 5) [48]; Taxonomic range: Nematoda: *Ascaris, Caenorhabditis*; Mollusca: Bivalvia; Polyplacophora. Arthropoda/Crustacea: *Artemia*; Arthropoda/Insecta: *Drosophila; Locusta migratoria *(migratory locust), *Apis mellifera *(honeybee).

3 Magenta: 'The Ascidian Mitochondrial Code' (NCBI  transl_table = 13) [48]. Taxonomic range: Urochordata: Tunicates.

4.Green: 'The Vertebrate Mitochondrial Code' (NCBI transl_table = 2) [49]; Taxonomic range: Vertebrata.

5.Cyan: 'The Yeast Mitochondrial Code' (NCBI transl_table = 3) [50]; Taxonomic range: *Saccharomyces cerevisiae, Candida glabrata, Hansenula saturnus, and Kluyveromyces thermotolerans.*

6.Yellow: 'The Ciliate, Dasycladacean and Hexamita Nuclear Code' (NCBI transl_table = 6) [51]. Taxonomic range: Ciliata: Oxytricha and Stylonychia, Paramecium, Tetrahymena, Oxytrichidae and probably Glaucoma chattoni. Dasycladaceae: Acetabularia and Batophora. Diplomonadida: *Hexamita inflata, Diplomonadida *ATCC50330 and ATCC50380.

7.Red: 'The Alternative Yeast Nuclear Code' (NCBI transl_table = 12) [52]. Taxonomic range: Endomycetales (yeasts): *Candida albicans, Candida cylindracea, Candida melibiosica, Candida parapsilosis, and Candida rugosa *(However, other yeasts, including *Saccharomyces cerevisiae, Candida azyma, Candida diversa, Candida magnoliae, Candida rugopelliculosa, Yarrowia lipolytica*, and *Zygoascus hellenicus*, definitely use the standard (nuclear) code).

## Unfolding and misfolding stabilities

We first study the direct effect of the eight different genetic codes on the average unfolding and misfolding stabilities (see Methods). Since we chose a neutral fitness landscape where mutations are either neutral or lethal, we expect that, independent of the mutation rate and the genetic code, the two folding stabilities will be close to the neutral thresholds, i.e. the minimum allowed stability values, which correspond to the maximum number of sequences, while larger stabilities correspond to many fewer sequences. However, large AT content (more than 50% AT) favors unfolding stability at the expense of misfolding stability, whereas small AT content (less than 50% AT) favors misfolding stability at the expense of unfolding stability. Consequently, for large AT content selection mainly acts on misfolding stability, which is expected to be closer to the neutral threshold, whereas unfolding stability is easily obtained and it is above the threshold. Conversely, for small AT content selection mainly acts on unfolding stability, which is expected to be close to its neutral threshold. In general, the smaller of the two stabilities is very close to the neutral threshold and almost independent of the genetic code, whereas the stability favored by the mutation process is above the neutral threshold, although this does not imply any gain in fitness, and it may depend on the genetic code and the mutation bias.

**Figure 3:**
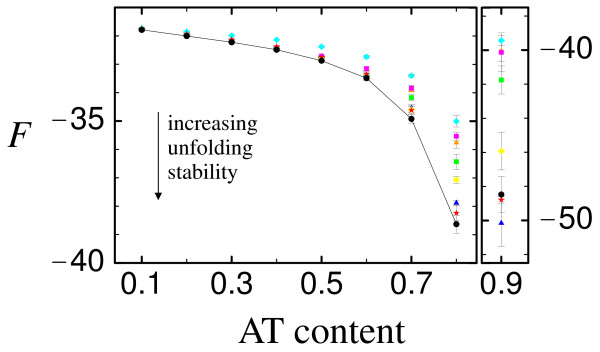
**Unfolding stability**. Average unfolding stability *F *vs AT content for the epsilon subunit of F1F0-ATP synthase (PDB id. 1aqt, chain A), exemplified for transition-to-transversion ratio *k *= 2 (the data for AT content 90% is shown using a different scale for better visibility). The standard genetic code is shown as black circle (which are connected by lines for better visibility), whereas the seven naturally occurring variants studied, as listed in Fig. 2, are shown using the following color scheme: (1, blue triangle): 'The Echinoderm and Flatworm Mitochondrial Code' (NCBItransl_table = 9) [48]; (2, orange triangle): 'The Invertebrate Mitochondrial Code' (NCBI transl_table = 5) [48]; (3, magenta square): 'The Ascidian Mitochondrial Code' (NCBIs transl_table = 13) [48]; (4, green square): 'The Vertebrate Mitochondrial Code' (NCBIs transl_table = 2) [49]; (5, cyan diamond): 'The Yeast Mitochondrial Code' (NCBIs transl_table = 3) [50]; (6, yellow diamond): 'The Ciliate, Dasycladacean and Hexamita Nuclear Code' (NCBIs transl_table = 6) [51]; (7, red star): 'The Alternative Yeast Nuclear Code' (NCBIs transl_table = 12) [52]. The error bars indicate the mean's standard deviation.

The behavior of the average unfolding stability *-F *for different genetic codes (as summarized in Fig. 2) is exemplified using the epsilon subunit of F1F0-ATP synthase (PDB id. 1aqt, chain A) and the transition-to-transversion ratio *k *= 2, see Fig. 3. The transition-to-transversion ratio *k *does not affect the results significantly, so that we omit the results for *k *= 20. As expected, there is hardly any influence of the genetic code on the average unfolding stability, except for extremely large AT biases (approx. 80% AT content or more) where unfolding stability is well above threshold and selection mainly acts on misfolding stability. In such cases, most alternative genetic codes display a smaller unfolding stability than the standard code (less negative *F*), but there are two exceptions, the 'Echinoderm and Flatworm Mitochondrial Code' (blue triangles) and the 'Alternative Yeast Nuclear Code' (red stars), which have slightly better unfolding stability than the standard code, even though the difference is minor and it does not imply any difference in fitness. The two other proteins we studied show a similar behavior (data not shown).

**Figure 4:**
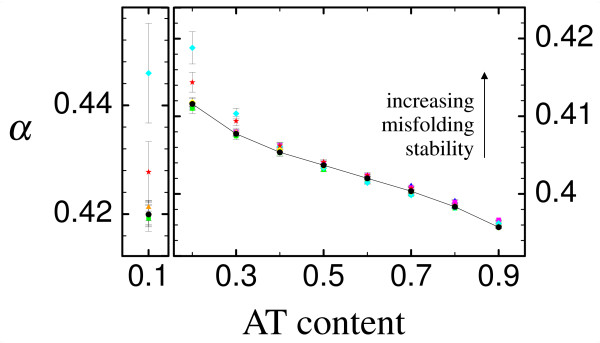
**Misfolding stability**. Average misfolding stability α vs AT content for the epsilon subunit of F1F0-ATP synthase (PDB id. 1aqt, chain A), exemplified for transition-to-transversion ratio *k *= 2 (the data for AT content 10% is shown using a different scale for better visibility). Symbols are as in Fig. 3, black circles indicate standard genetic code (which are connected by lines for better visibility), whereas the other different colors indicate the seven naturally occurring variants studied, as listed in Fig. 2. The error bars indicate the mean's standard deviation.

The behavior of the average misfolding stability *α *for the eight different genetic codes is likewise exemplified using the epsilon subunit of F1F0-ATP synthase (PDB id. 1aqt, chain A) and the transition-to-transversion ratio *k *= 2, see Fig. 4. Again, no significant difference can be noticed between transition-to-transversion ratio *k *= 2 and *k *= 20, so that we omit the latter. There is hardly any influence of the genetic code on the misfolding stability, significant differences are found only for very small AT content (approx. 20% AT content or less) at which misfolding stability is easily obtained. For such small AT content, most alternative genetic codes display an essentially identical unfolding stability, but there are two exceptions, the 'Yeast Mitochondrial Code' (cyan diamonds) and the 'Alternative Yeast Nuclear Code' (red stars), which yield larger misfolding stability than the standard code, even though the difference is minor. The two other proteins we studied show a similar behavior (data not shown).

We see from the two figures that the genetic code may influence the balance between unfolding and misfolding stability. For instance, the 'Yeast Mitochondrial Code' (cyan diamonds) yields systematically lower stability against unfolding (less negative *F*) when compared with other codes, and higher stability against misfolding at low AT, which is an effect similar to the one obtained by decreasing the AT content. Nevertheless, these differences are not relevant, since they are buffered at the level of fitness. In fact, we assume a neutral model in which the fitness is either ℱ = 0 or ℱ = 1 (in arbitrary units), so that differences in stability do not yield differences in fitness. Moreover, notice that the difference of, say, stability against unfolding between different codes is only significant at an AT content at which this stability is anyway high, i.e. it is far from the neutral threshold, so that the selective pressure mainly affects stability against misfolding. These differences might become relevant in a non-neutral fitness landscape where fitness depends smoothly on stability [44].

## Mutation and translation load

Next, we study the effect of the different genetic codes on mutation and translation loads (see Methods). These represent the fitness loss due to mutations and translation errors. The two loads differ by the rate at which a given error occurs and by the treatment of stop codons: In the case of mutation load, the rate of a mutation is given by the mutation process we use, which includes both the mutation bias and the transition-to-transversion ratio; mutations to stop codons are equivalent to mutations to sense codons as far as the chemical modification of the DNA sequence is concerned and hence included into the definition of the corresponding load (cf. Eq. (3) in Methods), and the associated fitness is zero. In the case of translation load, all mistranslation to sense codons are assigned equal rate. Since a premature end of translation by misinterpreting a sense codon as a stop codon is caused by release factors and not by tRNAs (and hence by a different mechanism than misinterpreting a sense codon as a another sense codon) which furthermore involves neighboring codons [53], for simplicity, we consider its error rate much smaller than the rate of missense errors in translation, and we neglect mistranslations to stop codons. In this way, the translation load is not explicitly influenced by the number of stop codons in the genetic code under consideration (cf. Eq. (4) in Methods).

**Figure 5:**
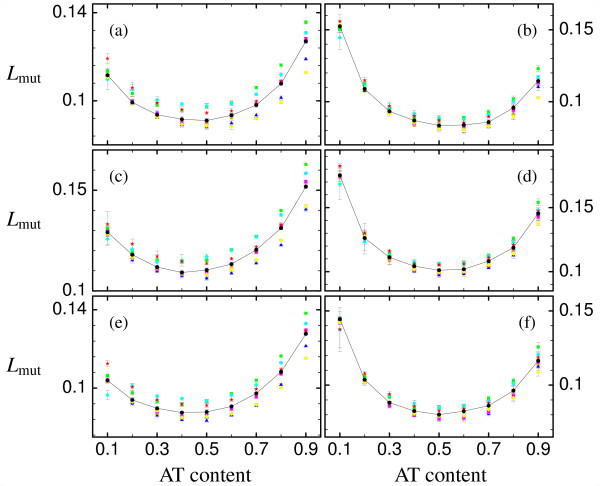
**Mutation load**. Average mutation load *L*_*mut *_vs AT content for (a),(b) for the epsilon subunit of F1F0-ATP synthase (PDB id. 1aqt, chain A) (c),(d) for the acyl carrier protein (PDB id. 1hy8, chain A) and (e),(f) for the cold-shock protein (PDB id. 1c9o, chain A) as well as for (a),(c),(e) transition-to-transversion ratio *k *= 2 and (b),(d),(f) *k *= 20. Symbols are as in Fig. 3, black circles indicate standard genetic code (which are connected by lines for better visibility), whereas the other different colors indicate the seven naturally occurring variants studied, as listed in Fig. 2. The error bars indicate the mean's standard deviation.

The average mutation load *L*_mut _for different genetic codes is exemplified using the three proteins described above, see Fig. 5. The left panels refer to transition-to-transversion ratio *k *= 2, while the right panels refer to *k *= 20, and the three rows refer to the three proteins. A large transition-to-transversion ratio usually yields smaller mutation loads, except for very small AT content (approx. 20% AT content or less) for which *k *= 20 increases the load considerably. In contrast to the unfolding and misfolding stabilities, different genetic codes show different mutation loads. This is in part due to the different form in which selection acts on stabilities and on loads in our model. Whereas unfolding and misfolding stabilities are strictly constrained in the fitness landscape of neutral or lethal mutations that we modelled, we assume that loads are not explicitly targeted by selection and are free to vary. Most of the alternative genetic codes display mutation loads larger than for the standard code. Nevertheless, some yield consistently smaller mutation loads in some range of mutation bias. For instance, the 'Yeast Mitochondrial Code' (cyan diamonds) yields a smaller mutation load for very small AT content (approx. 20% AT content), although it has a rather large load for large AT content which is characteristic of mitochondria genomes of Yeast (typically 75% to 85% AT content). Two alternative genetic codes, the 'Ciliate, Dasycladacean and Hexamita Nuclear Code (yellow diamonds) and the 'Echinoderm and Flatworm Mitochondrial Code' (blue triangles), display a smaller mutation load for large AT content.

**Figure 6:**
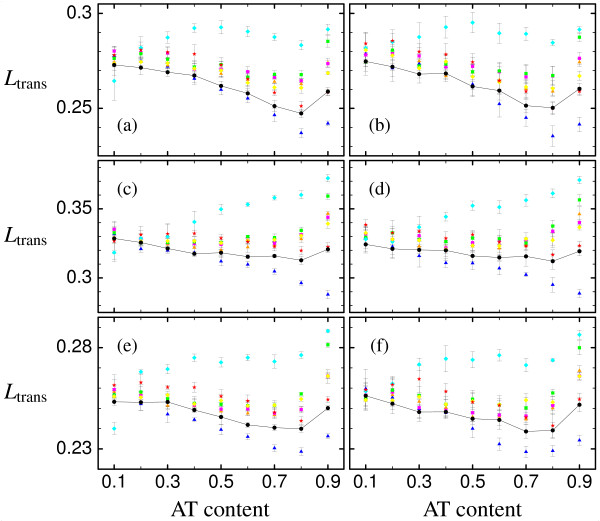
**Translation load**. Average translation load *L*_trans _vs AT content for (a),(b) for the epsilon subunit of F1F0-ATP synthase (PDB id. 1aqt, chain A) (c),(d) for the acyl carrier protein (PDB id. 1hy8, chain A) and (e),(f) for the cold-shock protein (PDB id. 1c9o, chain A) as well as for (a),(c),(e) transition-to-transversion ratio *k *= 2 and (b),(d),(f) *k *= 20. Symbols are as in Fig. 3, black circles indicate standard genetic code (which are connected by lines for better visibility), whereas the other different colors indicate the seven naturally occurring variants studied, as listed in Fig. 2. The error bars indicate the mean's standard deviation.

The behavior of the average translation load *L*_trans _for different genetic codes is likewise exemplified using the three proteins, see Fig. 6. Again, the left panels refer to transition-to-transversion ratio *k *= 2, while the right panels refer to *k *= 20, and the three rows refer to the three proteins. The different genetic codes show significantly different translation loads, and even different dependences on the mutation bias (decreasing or increasing translation load with increasing AT content). Notice that the dependence of the translation load on the bias is not due to how the error rates depend on the bias, as in the case of the mutation load, but it is due to how the mutation bias influences protein stabilities. Most alternative genetic codes yield a larger translation load than the standard code. Nevertheless, the 'Yeast Mitochondrial Code' (cyan diamonds) yields a smaller translation load for very small AT content (approx. 20% AT content or less), but this result does not hold for very large transition-to-transversion ratio and large AT content, both being characteristic of mitochondria genomes of Yeast. One alternative genetic code, the 'Echinoderm and Flatworm Mitochondrial Code' (blue triangles), results in a smaller translation load for most mutation biases, in particular for large ones, and independent of the transition-to-transversion ratio, and is hence preferential to the standard genetic code.

It is interesting to note that there seems not to be any trivial dependence of the average mutation load on the number of stop codons in the genetic code. Since the mutation load is calculated including mutations to stop codons, one would expect genetic codes containing more stop codons to have a larger mutation load than genetic codes containing fewer stop codons. This is, however, not the case, as one sees in Fig. 5 by comparing the 'Echinoderm and Flatworm Mitochondrial Code' (blue triangles) and the 'Yeast Mitochondrial Code' (cyan diamonds), which both have two stop codons, with the standard genetic code, which has three stop codons. Additionally, as our definition of translation load excludes mistranslations corresponding to stop codons (as misinterpreting a sense codon as a stop codon is caused by release factors and not by tRNAs and hence by a different mechanism than misinterpreting a sense codon as another sense codon), the lower translation load of the 'Echinoderm and Flatworm Mitochondrial Code' (blue triangles) in comparison to the standard genetic code seen in Fig. 6 is not trivially due to the fact that this alternative genetic code has two instead of three stop codons.

## Assumptions and empirical observations

Like all mathematical models of evolution, our model depends on several assumptions and parameters. An important assumption is that the population is genetically homogeneous, i.e. the product *Nμ *of population size times mutation rate is small. This assumption is considered approximately valid for eukaryotic and bacterial populations when considering an individual protein, in particular if population size is small. Strict validity of this assumption would imply that the number of different alleles at a typical locus is not larger than two. Despite that this is not the case, the number of alleles at a typical locus is usually small, so that the assumption is at least approximately valid. The high mutation rates of RNA viruses violate this assumption, and in this case recent work [54,55] has shown that even the neutral model should be re-formulated in the framework of the quasi species theory [56]. If we considered a whole evolving genome instead of a single protein, the approximation of very small mutation rate would not be justified, and a new interesting effect has to be considered, namely the hitch-hiking effect, which consists in the fixation of mildly disfavorable alleles driven by a positively selected allele present in the same chromosome. The mutation process was modelled using two parameters, the mutation bias and the transition-to-transversion ratio. While this parameterization might appear too simplified, it has the merit to focus on two variables whose relevance has been pointed out by a large number of experimental studies, statistical analysis, and models.

As evolutionary model, we adopt a model in spirit Kimura's neutral theory of molecular evolution [42,43], in which mutations are either neutral or lethal. The assumption of neutrality prevents us to study how the genetic code affects the fitness that can be achieved in evolution (in the neutral model the fitness of a viable sequence is equal to one in arbitrary units by definition), however it allows to study its influence on the mutation and translation load without any further assumptions concerning the shape of the fitness landscape. Whether a mutation is neutral or lethal is decided based on unfolding and misfolding stabilities of the resulting amino acid sequence.

The ingredient of our model that seems more debatable is the genotype to phenotype mapping, which is based on predicted unfolding and misfolding stabilities. While we do not claim that our predictions are accurate in individual cases, our experience suggests that they are statistically correct, so that they are able to discover statistical trends, which is what we address here. However, a limitation of our approach consists in that the mitochondrial proteome mainly contains membrane proteins, whereas our predictions of unfolding and misfolding stabilities are only valid for soluble (i.e., non-membrane) proteins, hence implying caution about the interpretation of the deleterious effect of mitochondrial codes. Besides of that, we note that the statistical potentials that we use here are quite general, as they have been optimized based on all soluble globular proteins in the protein data bank, and they are not limited to a particular organism or protein family. An alternative way to derive empirical potentials for protein evolution consists in fitting the potentials to maximize the likelihood of the observed sequences, which provides an improved fit in an evolutionary context [57].

Another important point concerns the choice of the neutral thresholds *α*_thr _and *F*_thr_.We have tested in previous studies that changing the neutral thresholds within reasonable limits (approx. 25% in both directions) does not significantly affect the results of neutral simulations [28-32].

Furthermore, we assume in our model that all synonymous mutations that do not change the amino acid sequence are neutral. Nevertheless, it is known that the use of alternative codons has important phenotypic effects on the translation dynamics [58,59], and it can affect the rate of translation errors [60]. Modelling these effects, however, would require assumptions on the abundance of different tRNA molecules and the dynamics of the ribosome that are outside the scope of our model. Therefore, selection on optimal codon usage is a way to reduce the load due to translation error that is complementary to the influence of the genetic code that we investigate here.

Finally, an interesting empirical observation that might be related with our protein evolution model is the finding that long genes tend to have lower codon usage bias [61]. One of us previously observed that longer proteins have contact interaction energies that are less optimized than for shorter proteins. This finding has a simple neutral explanation, since the number of contact interaction per protein is larger in longer proteins, whereas the conformation entropy loss per residue that these interactions have to compensate does not depend on protein length [62]. Therefore, contact interactions are subject to weaker selective constraints in long proteins. If this is true, and if the selective forces on codon bias are mainly due to the advantage of reducing folding problems in mistranslated proteins, which is now considered the prevailing view [14], one would expect that the selective forces on codon bias are also reduced for longer proteins, consistent with the empirical observation by Duret and Mouchiroud [61]. This subject may be addressed in the framework of an improved model in which fitness takes into account both the two protein folding stabilities and the translation load.

## Conclusions

Our results show that the standard genetic code is generally preferable to naturally occurring variants, in the sense that it typically yields smaller mutation and translation loads. This finding is consistent with the view that the standard genetic code is well adapted for reducing the consequences of translation errors on protein folding stability, as expected in the framework of the adaptive theory on the origin of the genetic code, and that the fixation of alternative genetic codes is either a slightly deleterious event that is mutationally driven or, if it brings some selective advantage, this is through the reduction of the number of tRNA needed.

Nevertheless, we found one alternative genetic code (the 'Echinoderm and Flatworm Mitochondrial Code') that seems to be better at reducing mutation and translation loads, and particularly yields better translation load except for very small AT content (approx. 20% AT content or less). As translation load is a very important constraint in protein evolution [14], this difference might result in small but significant fitness differences and hence be subject to positive selection. Therefore, although our model confirms the view that code changes are slightly deleterious events in the majority of the cases, it also suggests that adaptation cannot be excluded for one of the studied cases.

## Methods

### Unfolding and misfolding stability

As in our previous work [28-32], the unfolding free energy *F*(**A**) of a protein with sequence **A **= {*A*_1_...*A*_*L*_} and contact matrix *C*_*ij*_= 1 if the minimal interatomic distance between residues *i *and *j *is below 4.5 Å, 0 otherwise, is defined as(1)

where *U*(*a*, *b*) is the contact interaction matrix determined in Ref. [27]. Although rather simple, this model is accurate enough to allow quantitative predictions of the folding free energy of small proteins that fold with two-state thermodynamics (the correlation coefficient between experimental and predicted free energy is *r *= 0.92 over a representative test set of 20 proteins, UB, unpublished result) and of the stability effect of mutations (correlation coefficient *r *= 0.72 over a set of 195 mutations, UB, unpublished result). This is comparable to state-of-the-art programs such as Fold-X [63]. However, the computational simplicity of the model makes it affordable to use it for simulating very long evolutionary trajectories, which would not be possible using other tools. The unfolding free energy should also take into account the loss of conformation entropy upon folding, which we modelled in other works as *sL *with *s *being the chain entropy per amino acid and *L *the protein length. However, this term only induces a constant shift *sL *in the unfolding free energy, *F*'(**A**) = ∑_*i<j*_*C*_*ij*_*U*(*A*_*i*_, *A*_*j*_) + *sL *and its effect is just to shift the neutral threshold *F*_thr _in the same direction, without influencing the results.

The normalized energy gap *α*(**A**) measures the (positive) energy difference between alternative compact conformations and the native conformation, and it is defined using the random energy model [64,65] as(2)

with *A *= 0.1, *B *= 4, *q*_0 _= 0.1, and *N*_*c *_= ∑_*i*<*j *_*C*_*ij*_. ⟨*e*⟩_**A **_and *σ*_*e*,**A **_are the mean and standard deviation of the interaction energy of both native and non-native contacts in sequence **A.**

### Mutation process

Mutations are modelled through the HKY process [66], in which the mutation rate from nucleotide *n *to *n*', *T*(*n*, *n*'), is *μf *(*n*') if *n*→*n*' is a transition, and *μkf*(*n*') if it is a transversion. The transition-to-transversion ratios used in this work are *k *= 2 and *k *= 20, suitable for nuclear and mitochondrial DNA, respectively [46,47]. The microscopic rate *μ *is assumed to be very small and it does not affect the results. We further assume π(A) = π(T) and π(C) = π(G) (Chargaff second parity rule), so that the only parameter of the mutation model is the stationary AT frequency, AT = π(A) + π(T).

### Simulation of the evolutionary process

Our model of protein evolution cannot be treated analytically, so that we have to study it using numerical simulations (see Fig. 1). We start each simulation with the native amino acid sequence obtained from the Protein Databank (PDB) of the chosen structure, from which we construct a corresponding 'native' DNA sequence by randomly choosing codons using the genetic code under consideration with weights determined by the given AT content (inverse translation). The simulation thus starts as close as possible to the equilibrium with the chosen AT content. The initial part of the trajectory is discarded to ensure that relevant quantities are sampled at the stationary state. To do so, we visually verified that the stabilities had reached the stationary state for simulations with the standard code, and then discarded the same transient part of the trajectory for all alternative codes.

The simulations are performed as follows. At every step, we randomly select one DNA site *j *with probability dependent on the nucleotide *n*_*j *_occupying it,  and we extract the mutated nucleotide *n*' ≠ n_*j *_with probability proportional to *T*_*μ*_(*n*_*j*_,*n*'). The mutated DNA is then translated to an amino acid sequence, whose unfolding and misfolding stabilities are computed through Eqs. (1) and (2). The mutation receives fitness ℱ = 1 (in arbitrary units) and is accepted if both *F *<*F*_thr _and *α *>*α*_thr_, or gets a fitness ℱ = 0 and is rejected otherwise, hence assuming a model in spirit of Kimura's neutral theory of molecular evolution. Mutations to stop codons are considered lethal and receive a fitness ℱ = 0. As the mutation process is continuous, the waiting time until a new mutation arises is a Poissonian variable with mean *μ*^-1^. Instead of drawing an explicit waiting time for each mutation to arise, we assign each mutation the mean time *μ*^-1 ^(this is equivalent to performing an average over possible realizations of waiting times). In case several mutations occur before one gets fixed, the weighting of the sequence before the accepted mutation is increased accordingly. The simulation is run for a large number of 10^6 ^mutations to obtain long evolutionary trajectories which are used to calculate the averages. The neutral thresholds *F*_thr _and *α*_thr _are calculated for each simulated protein and kept fixed during the simulations. We set *F*_thr _= γ*F*_nat _and *α*_thr _= *γα*_nat_, where *F*_nat _and *α*_nat _are the unfolding free energy and misfolding stability of the respective native amino acid sequence. The factor *γ *is chosen as *γ *= 0.98, so that the native sequence is considered viable. Changing *γ *within reasonable limits (approx. 25% in both directions) does not significantly effect the results.

### Mutation and translation load

The mutation load per site *L*_mut _(**n**) of a DNA sequence **n **translated to amino acid sequence **A**[**n**] is defined as(3)

where Δℱ(**A**[**n**] → **A**[**n'**]) is the fitness difference between amino acid sequence **A**[**n**'], as translated from the mutated DNA sequence **n**', and amino acid sequence **A**[**n**], and *R*_mut_(n→n') is the rate of a mutation from **n **to **n**', which is calculated according to our mutation process with the mutation rate μ and only single nucleotide mutations are considered (i.e. only terms linear in μ, ignoring the higher order terms which have rates proportional to μ^2 ^and μ^3 ^and are hence much smaller than *μ*). Since we study a neutral model, so that the fitness of a viable sequence is ℱ = 1 (in arbitrary units) and ℱ = 0 otherwise, the fitness difference Δℱ(**A**[**n**] → **A**[**n'**]) can only take the values 0 if **A**[**n**'] is a viable sequence as well, and 1 if **A**[**n**'] is not a viable sequence. As we restrict ourselves to those **n**' which differ from **n **by a single nucleotide, the sum in Eq. (3) contains 9*L *terms, and hence the normalization *N*_mut_(**n**) ≡ ∑_**n' **_Θ[*R*_mut_(**n **→ **n'**)], where Θ[*R*_mut_(**n **→ **n'**)] = 1 if *R*_mut_(**n**→**n**')> 0 and 0 otherwise, yields *N*_mut_(**n**)= 9*L *independent of **n**. Consequently, in the extreme case of all DNA sequence **n**' which differ from **n **by a single nucleotide (one point mutation) being viable and hence Δℱ(**A**[**n**] → **A**[**n'**]) = 0 for all these **n**', the mutational load is *L*_mut _(**n**) = 0. If the transition-to-transversion ratio was *k *= 1 so that *R*_mut_(**n**→**n**') = *μ *for all **n**' differing from **n **by a single nucleotide, then μ*N*_mut _= ∑_**n' **_*R*_mut _(**n **→ **n'**) and the mutation load *L*_mut _(**n**) would be the fraction of lethal sequences **n^'^**. Due to our choice for the normalization, there is no explicit dependence of *L*_mut_(**n**) on sequence length.

The translation load per site *L*_trans_(**n**) of a DNA sequence **n **translated to amino acid sequence **A**[**n**] is similarly defined as(4)

where *R*_trans _(**A**[**n**] → **A**[**n'**]) is the rate of a translation error resulting in amino acid sequence **A**[**n**'] instead of **A**[**n**] and *v *the rate of single nucleotide mismatches. For simplicity, we assume that *R*_trans_(n→n') = *v *if nucleotide sequence **n**' resulted from **n **by a single nucleotide mismatch and does not contain any stop codon and 0 otherwise (i.e. only terms linear in *v *are considered, as for the mutation load), so that our definition of the translation load does not depend on the error rate of translation, which is approximately 10^-4 ^per translated mRNA codon [67] but may differ from species to species. We exclude nucleotide sequences **n**' containing a stop codon for the computation of the translation load since a premature end of translation by misinterpreting a sense codon as a stop codon is caused by release factors and not by tRNAs (and hence by a different mechanism than misinterpreting a sense codon as another sense codon) which furthermore depends on neighboring codons [53]. For simplicity, we neglect here this error rate with respect to the rate of missense errors in translation. In this way, the translation load does not explicitly dependent on the number of stop codons, and the normalization *N*_trans_(**n**) = ∑_**n' **_Θ[*R*_trans_(**n **→ **n'**)], where Θ[*R*_trans_(**n **→ **n'**)] = 1 if *R*_trans_(**n **→ **n'**) > 0 and 0 otherwise, does dependent on **n**. Even though we use the above general definition, Eq. (4), in analogy to the mutation load, note that with our choice for *R*_trans_(**n **→ **n'**), *νN*_trans _= ∑_**n' **_*R*_trans_(**n **→ **n'**) and the translation load *L*_trans_(**n**) is the fraction of lethal sequences among all **n**' differing from **n **by a single nucleotide and not containing a stop codon. Due to our choice for the normalization, there is neither an explicit dependence of *L*_trans_(**n**) on sequence length nor on the number of stop codons in the genetic code considered.

### Protein list

We studied the three following proteins structures: (i) the epsilon subunit of F1F0-ATP synthase (PDB id. 1aqt, chain A, *α *+ *β *protein, 135 amino acids, GenBank CBG36944.1), (ii) the acyl carrier protein (PDB id. 1hy8, chain A, all-*β *protein, 76 amino acids, GenBank BAA10975.1 and CAB13465.1), and (iii) the cold-shock protein (PDB id. 1c9o, chain A, all-β protein, 66 amino acids, GenBank CAA51790.1).

## Authors' contributions

MP and UB designed research; SGS performed research; SGS, UB, and MP analyzed data; MP and UB wrote the paper. All authors read and approved the final manuscript
